# Supplementary material for: Quantitative Trait Loci (QTL) Analysis of Seed Protein and Oil Content in Wild Soybean (Glycine soja)
Source: Int J Mol Sci. 2023 Feb 17;24(4):4077. doi: 10.3390/ijms24044077 (PMC9959443; doi:10.3390/ijms24044077)
Supplement: Supplementary file 1 [file ijms-24-04077-s001.zip › ijms-2217991-supplementary.pdf]

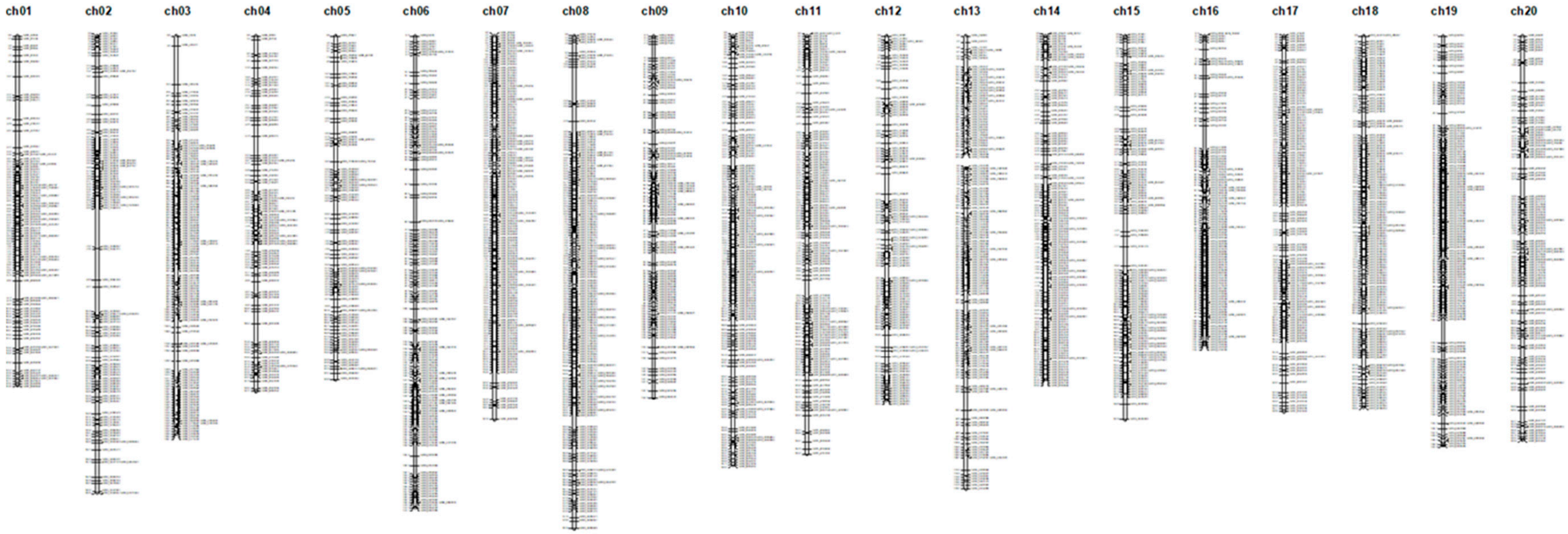

Figure S1. Genetic linkage map for the F_2 population derived from a cross between Daepung and GWS-1887.

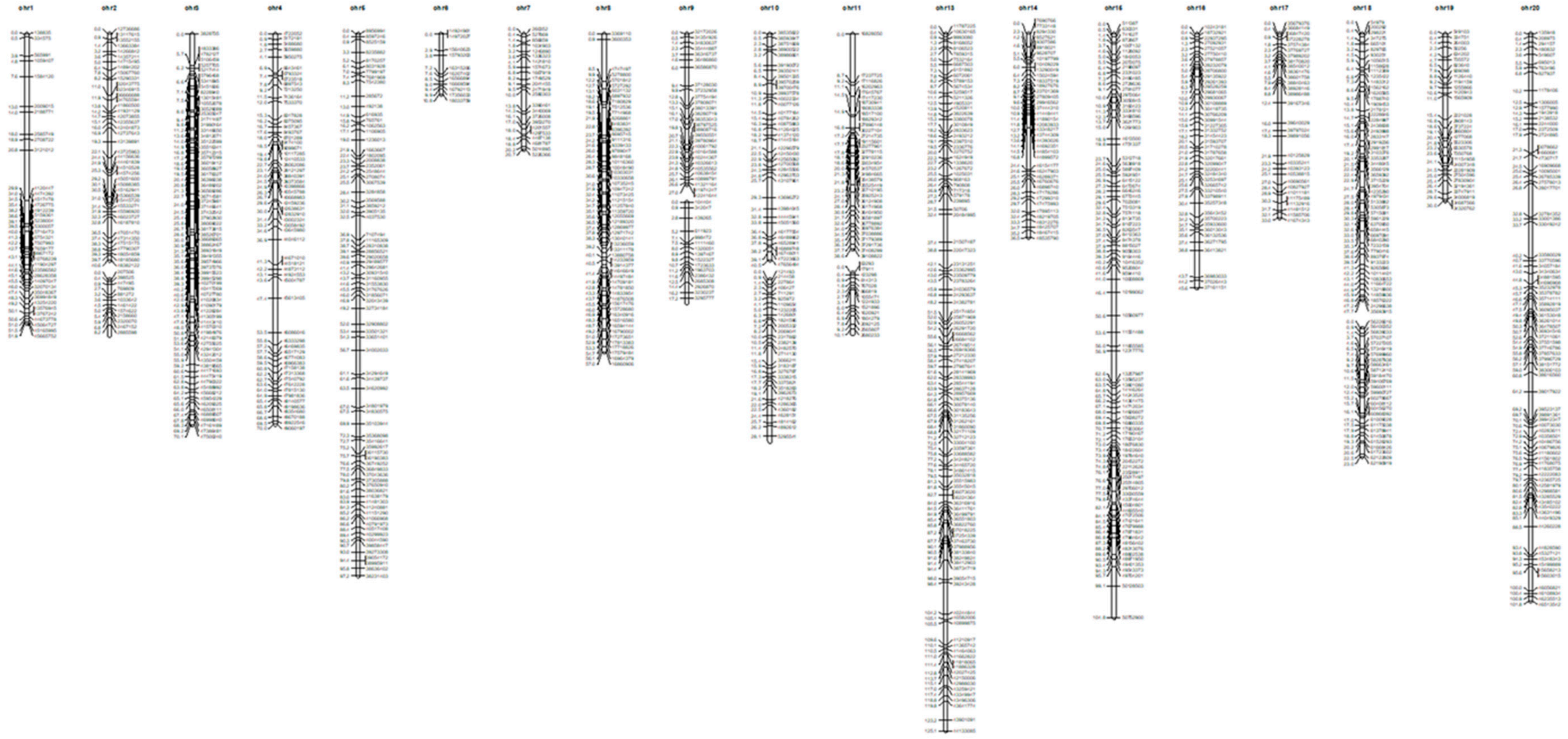

Figure S2. Genetic linkage map for the BC_1F_2 population derived from a cross between Daepung and GWS-1887.
